# Field-Scale Estimation of Maize Leaf Area Index from UAV Multispectral Imagery Using Compact Spectral and Texture Predictors

**DOI:** 10.3390/s26196008

**Published:** 2026-09-22

**Authors:** Yu Zhang, Yanying Bai, Hongxin Li, Yang Xu, Zimao Chen, Jiahua Yan, Yongmei Wang

**Affiliations:** 1 College of Water Conservancy and Civil Engineering, Inner Mongolia Agricultural University, Hohhot 010018, China; 2293891413@emails.imau.edu.cn; 2 National Key Laboratory of Eco-Environmental Effects of Water Engineering in Arid Areas, Inner Mongolia Agricultural University, Hohhot 010018, China; 3 Yichang Sub-Center of Water Resources Development Research Center of Inner Mongolia Hetao Irrigation District, Bayannur 015100, China

**Keywords:** UAV remote sensing, maize, leaf area index, spectral–texture features, precision agriculture, spatial heterogeneity

## Abstract

Leaf area index (LAI) is an important indicator of canopy development and is widely used for crop diagnosis and field-scale monitoring. We developed a UAV multispectral approach to estimate and map maize LAI in Tumed Right Banner, Inner Mongolia, China. The dataset comprised 488 paired observations from 122 georeferenced locations in 13 fixed plots, measured repeatedly at four growth stages. Vegetation indices and gray-level co-occurrence matrix texture features were derived from the green, red, red-edge, and near-infrared bands. Five models were evaluated using a single 70:30 split. Model robustness was further examined through 100 repeated stage-stratified splits, leave-one-plot-out validation, leave-one-growth-stage-out validation, and RF-based feature ablation. In the single-split benchmark, RF achieved R^2^ = 0.889, RMSE = 0.249, and RRMSE = 0.082. Across the repeated splits, the corresponding values were R^2^ = 0.876 ± 0.016, RMSE = 0.258 ± 0.018, and RRMSE = 0.085 ± 0.006. Leave-one-plot-out validation gave R^2^ = 0.878 ± 0.062, whereas leave-one-growth-stage-out testing revealed weaker, stage-dependent transferability (RF R^2^, −0.542 to 0.441). Feature ablation showed that vegetation indices provided most of the predictive information; adding the two NIR texture variables increased R^2^ by 0.010 relative to vegetation indices alone. Because the field platform lacked independent plot replication, the maps are interpreted as descriptions of canopy heterogeneity rather than evidence of treatment effects. The approach is suitable for site-specific maize canopy monitoring, but broader application requires independent validation across years and locations.

## 1. Introduction

Leaf area index (LAI) describes canopy structure, light interception, and the crop growth status [1]. Destructive sampling and ground optical instruments can provide reliable point measurements, but both are labor-intensive and offer limited spatial coverage. These constraints make continuous, non-destructive field-scale monitoring difficult. Low-altitude unmanned aerial vehicles (UAVs) equipped with lightweight sensors offer a practical alternative for crop phenotyping and field monitoring [2,3]. Compared with satellite data, UAV multispectral imagery provides finer spatial resolution and flexible acquisition times, which are useful for resolving canopy variability within field experiments.

Vegetation indices are commonly used to estimate LAI because they reflect canopy greenness, chlorophyll absorption, and vegetation cover [4]. Their performance can, however, be affected by soil background, canopy architecture, and saturation under medium-to-high canopy cover [5,6,7]. High-resolution UAV imagery also contains information on row structure, canopy closure, and texture. Texture metrics, commonly derived from gray-level co-occurrence matrices (GLCMs) [8], can therefore complement spectral indices by representing the canopy spatial structure. Previous studies have reported improved LAI estimates when spectral and texture information are combined, although the gain depends on crop type, sensor configuration, feature set, and validation design [9,10,11,12,13].

LAI estimation has progressively moved from empirical regression toward machine learning and deep learning approaches [14,15]. Random forest, gradient boosting, and neural networks can accommodate nonlinear relationships between spectral–texture predictors and LAI. Their apparent accuracy, however, remains sensitive to sample size, feature dimensionality, input structure, and the degree of independence between training and testing data [3,16,17,18].

UAV-based maize LAI estimation using multispectral features, texture metrics, and machine learning models has been widely reported [3,11,17,18]. The contribution here is therefore not the first application of spectral–texture predictors. Instead, the work presents a cautious field-scale workflow built on three vegetation indices and two texture variables, separates a single-split benchmark from repeated and group-aware validation, quantifies the added value of texture through feature ablation, and restricts map interpretation to descriptive canopy heterogeneity. This framework helps avoid overinterpreting accuracy derived from one site, one season, and non-replicated fixed plots.

UAV multispectral imagery and synchronous ground LAI measurements were collected from a maize field in Tumed Right Banner, Baotou, Inner Mongolia, China. The objectives were to: (1) construct vegetation-index and gray-level co-occurrence matrix (GLCM) texture candidates from the green, red, red-edge, and near-infrared bands and identify a compact predictor set for maize LAI estimation; (2) compare RF, SVR, XGB, MLP, and 1D-CNN using the same inputs and examine feature contributions with SHAP; and (3) map LAI at the jointing, tasseling, silking, and grain-filling stages to describe field-scale canopy heterogeneity relevant to crop diagnosis and management zoning. The field was part of an existing soil fertility improvement platform. Each management background was represented by one fixed plot, and some plots received additional nitrogen. Plot-level LAI differences were therefore treated as descriptive spatial patterns and not as independent causal effects of tillage, straw return, or nitrogen application.

## 2. Materials and Methods

### 2.1. Study Area

The field experiment was located at the Science and Technology Park of the Vocational and Technical College of Inner Mongolia Agricultural University in Tumed Right Banner, Baotou, Inner Mongolia, China (40°59′ N, 110°56′ E). The location of the study area and the layout of the fixed plots are shown in Figure 1. The soil is predominantly sandy loam, and the area has a temperate continental monsoon climate. Mean annual air temperature is approximately 6–8 °C, annual precipitation is about 400 mm, the frost-free period is about 140 days, and the elevation is approximately 1015 m. Maize is the dominant crop.

### 2.2. Experimental Design

The experiment was conducted on an existing soil fertility improvement platform. The 13 fixed management backgrounds were treated as observational plot groups rather than independent treatment replicates. Some groups received additional nitrogen, and the number of georeferenced sampling locations differed among groups. Accordingly, plot-level contrasts were used only to describe spatial heterogeneity.

The experimental area contained 13 fixed plots: deep tillage without straw return (DT1), deep tillage with straw return (DT2), deep tillage with straw return plus additional nitrogen (DT2-N), subsoiling without straw return (SB1), subsoiling with straw return (SB2), subsoiling with straw return plus additional nitrogen (SB2-N), strip rotary tillage without straw return (SDR1), strip rotary tillage with straw return (SDR2), no-tillage without straw return (NT1), no-tillage with straw return (NT2), no-tillage with straw return plus additional nitrogen (NT2-N), farmer shallow rotary tillage without straw return (CK1), and farmer shallow rotary tillage without straw return plus additional nitrogen (CK1-N). Plots labeled “-N” received additional nitrogen; their LAI patterns cannot therefore be attributed to tillage or straw-return practices alone.

The maize cultivar was Xianyu 696. Each plot was 40 m long, with a 0.6 m row spacing and a planting density of 82,500 plants ha^−1^. All plots received the same basal fertilizer rates: 225 kg N ha^−1^, 210 kg P_2_O_5_ ha^−1^, and 202.5 kg K_2_O ha^−1^. Diammonium phosphate (18% N and 46% P_2_O_5_) and potassium sulfate (51% K_2_O) were applied before planting. Urea (46% N) was dissolved in the fertigation tank and applied with drip irrigation at the jointing, large-trumpet, and grain filling stages in a 3:6:1 ratio. The DT2-N, SB2-N, NT2-N, and CK1-N plots received an additional 300 kg ha^−1^ urea, equivalent to 138 kg N ha^−1^, giving a total N rate of 363 kg ha^−1^. Four irrigations were applied at the jointing, large-trumpet, silking, and grain-filling stages, each supplying 750 m^3^ ha^−1^.

The platform did not include independent plot replication. LAI comparisons among the fixed plots were therefore limited to descriptive spatial analysis; no significance tests were performed, and the results were not used to rank the independent agronomic effects of tillage, straw return, or nitrogen application.

### 2.3. Ground LAI Measurements

Ground LAI was measured at jointing, tasseling, silking, and grain filling on 21 June, 21 July, 14 August, and 23 August 2025, respectively. On each date, 122 georeferenced 1 m × 1 m quadrats were assessed across the 13 plots. The same locations were revisited at all stages, producing 488 paired observations. All quadrats sampled on a given date were assigned the same field-level growth-stage label. Because each plot contained 6–26 sampling locations per stage, plot identity was retained for leave-one-plot-out validation. Three representative maize plants were selected in each quadrat. Fully expanded leaves remained attached while leaf length and maximum width were measured with a tape measure. Individual leaf area was calculated as length × maximum width × 0.75, a commonly used coefficient for fully expanded maize leaves [19]. Quadrat-scale LAI was calculated from planting density. For reproducibility, all analyses used LAI-Z-SJ.xlsx; the LAI column supplied the response variable and the five predictor columns supplied the model inputs:

(1) $$\mathrm{LAI}=\frac{K\cdot D}{M\cdot S}\sum_{j=1}^{M}\sum_{i=1}^{N_{j}}\left(L_{\mathrm{ij}}\times W_{\mathrm{ij}}\right)$$

where *LAI* is the leaf area index; *K* is the maize leaf-area correction coefficient (0.75) for fully expanded leaves [19]; *D* is planting density (plants m^−2^); *M* is the number of measured plants per quadrat (3); *N*_j_ is the number of fully expanded leaves on the j-th plant; *L*_ij_ and *W*_ij_ are the length and maximum width (cm) of the *i*-th leaf on the *j*-th plant; and S is the area conversion coefficient (10,000 cm^2^ m^−2^). Ground measurements and UAV image acquisition were completed on the same dates and were used to build and evaluate the LAI models.

### 2.4. UAV Multispectral Data Acquisition and Preprocessing

Multispectral images were acquired with a DJI Mavic 3 Multispectral UAV (DJI, Shenzhen, China) equipped with one RGB camera and four single-band multispectral sensors. The central wavelengths of the green (G), red (R), red-edge (RE), and near-infrared (NIR) bands were 560, 650, 730, and 860 nm, respectively. The G, R, and RE bandwidths were 32 nm, while the NIR bandwidth was 52 nm (±26 nm). UAV flights and ground LAI measurements were completed on the same dates. Flights were conducted between 10:00 and 13:00 local time under clear or slightly cloudy conditions with low wind speed. Flight height was 50 m, flight speed was 5 m s^−1^, and forward and side overlaps were both 80%. Images were collected along predefined routes at the four growth stages.

A standard reflectance calibration panel was photographed before each flight to improve reflectance consistency among dates, and its information was used during radiometric correction. DJI RTK positioning supported image mosaicking, orthorectification, and quadrat matching. Images were processed in DJI Terra (version 4.5.0; DJI, Shenzhen, China) for alignment, aerial triangulation, mosaicking, orthorectification, and radiometric correction. Independent check-point data were unavailable, so orthomosaic positional accuracy was not quantified with an RMSE; the manuscript therefore reports the RTK-supported georeferencing workflow without claiming independently verified orthomosaic accuracy. The four calibrated orthomosaics were then used to derive vegetation indices and texture features from the G, R, RE, and NIR bands.

Before feature extraction, plot boundaries were delineated from the orthomosaics and field layout. Plot edges, field roads, obvious bare soil, shadows, and abnormal pixels were excluded to reduce non-crop background effects. Quadrat centers were matched to the UAV orthomosaics, and mean spectral-index and texture-feature values were extracted for the corresponding quadrat areas. These values were paired with synchronous ground LAI measurements to form the modeling dataset. Because the number of quadrats was limited, quadrat-scale observations may not fully represent within-plot canopy variability; this limitation was considered when interpreting the maps.

### 2.5. Vegetation Indices and Texture Features

#### 2.5.1. Vegetation Indices

Candidate variables were derived from the calibrated G, R, RE, and NIR orthomosaics. Vegetation indices represented canopy spectral responses, whereas texture metrics represented spatial variation in canopy structure. Both groups were considered during feature screening and model development [3,11].

Vegetation indices combine spectral bands to represent canopy greenness, vegetation cover, chlorophyll-related responses, and soil background effects, and are widely used for LAI estimation [4]. Soil-adjusted indices can reduce background interference before canopy closure [5]. MCARI-type indices and related combinations are sensitive to green leaf area [6], while red-edge indices can help characterize LAI and chlorophyll variation at medium-to-high canopy cover [7]. Using the bands available from the DJI Mavic 3 Multispectral sensor, we calculated soil-adjusted, red-edge-sensitive, combined-enhancement, normalized-difference/ratio, and auxiliary indices (Table 1).

#### 2.5.2. Texture Feature Extraction

Texture metrics were extracted to represent the canopy spatial structure not fully captured by vegetation indices. Such metrics describe spatial variation in the canopy and can complement spectral variables [9,10,11,12,13]. We used the gray-level co-occurrence matrix (GLCM), a widely applied image texture method [8]. For a specified distance and direction, the GLCM summarizes co-occurrence relationships among pixel gray levels and thereby characterizes local uniformity, contrast, and complexity.

The G, R, RE, and NIR images were used for texture calculation. The workflow was implemented in Python (version 3.11.10) with NumPy (version 1.26.4) and the scikit-image (version 0.25.2) GLCM routines graycomatrix and graycoprops [20]. A 5 × 5 pixel moving window was used; gray levels were quantized to 16 levels, pixel distance was set to 1, and GLCMs were calculated in four directions (0°, 45°, 90°, and 135°). Statistics from the four directions were averaged for each band.

Eight texture metrics were derived for each band: mean (MEAN), variance (VAR), homogeneity (HOM), contrast (CON), dissimilarity (DIS), entropy (ENT), angular second moment (ASM), and correlation (COR), yielding 32 candidate texture variables. Studies have shown that texture can improve UAV-based LAI estimation when combined with spectral variables, although its contribution is context-dependent [9,10,11,12,13]. Texture metrics and vegetation indices were therefore considered jointly during predictor selection and model development.

### 2.6. Feature Selection and Model Interpretation

Feature selection was intended to identify a compact predictive set, not a group of independent explanatory variables. Candidate vegetation indices and texture metrics were first assessed using their Pearson correlations with measured LAI and their physical relevance. Three vegetation indices (MCARI1_GOSAVI, GSAVI, and MCARI1_MRETVI) and two NIR texture metrics (NIR_HOM and NIR_ENT) were retained before model benchmarking. No VIF threshold was imposed at this stage. Pairwise correlations and variance inflation factors (VIFs) were calculated after selection to make shared information explicit. Correlations with LAI were 0.903 for MCARI1_GOSAVI, −0.766 for GSAVI, 0.818 for MCARI1_MRETVI, −0.660 for NIR_HOM, and 0.684 for NIR_ENT. Several predictors were strongly correlated, and VIFs ranged from 10.51 to 31.54. The five variables are therefore treated as a predictive feature set rather than as independent explanatory variables.

SHAP (SHapley Additive exPlanations; version 0.48.0) values were calculated for the RF model to examine the relative contribution of the selected predictors [21]. Mean absolute SHAP values were used to summarize their contribution to RF predictions. Because SHAP values are model specific and correlated predictors can redistribute importance among related variables, held-out permutation importance was also calculated for RF and SVR. Interpretation focused on the relative contribution of vegetation-index and texture-feature groups, not on causal effects of individual variables.

### 2.7. LAI Estimation Models and Accuracy Metrics

All models were developed with the same three vegetation indices and two NIR texture metrics: MCARI1_GOSAVI, GSAVI, MCARI1_MRETVI, NIR_HOM, and NIR_ENT. The vegetation indices primarily represented canopy spectral responses, whereas NIR_HOM and NIR_ENT contributed complementary information on canopy spatial structure.

Five models were evaluated: random forest (RF), support vector regression (SVR), extreme gradient boosting (XGB), multilayer perceptron (MLP), and one-dimensional convolutional neural network (1D-CNN). RF, SVR, and XGB are established nonlinear regression algorithms [22,23,24], while MLP and 1D-CNN can represent nonlinear relationships in structured inputs [14,15]. Previous LAI studies show that model performance depends on sample size, predictor type, and model structure [3,16,17,18]. The five methods were therefore compared as benchmarks under the same five-input setting rather than presented as separate methodological innovations.

The 1D-CNN was not trained end to end on raw image patches. It received the same five structured predictors used by the other models and was implemented as a one-dimensional convolutional regression model. It was included as a neural-network benchmark rather than as the primary methodological contribution.RF and SVR were implemented using scikit-learn (version 1.7.1), XGB using XGBoost (version 3.0.1), and MLP and 1D-CNN using PyTorch (version 2.2.0+cpu).

RF used 320 trees, a maximum depth of 7, a minimum of 10 samples per leaf, a minimum of 22 samples for node splitting, and a maximum feature proportion of 0.40. SVR used a radial basis function kernel with C = 0.30, epsilon = 0.35, and gamma = 0.12. XGB used 180 trees, a maximum depth of 2, and a learning rate of 0.04. To limit overfitting, its subsampling and column-sampling ratios were both 0.65; minimum child weight was 10, gamma was 0.35, and L1 and L2 regularization parameters were 1.0 and 6.0, respectively. These settings were fixed before final testing on the basis of preliminary exploration of the training data and published parameter ranges. We did not perform an exhaustive grid search, and no held-out test observations were used to select settings.

The 1D-CNN contained two one-dimensional convolutional layers, each with a kernel size of 2 and with 32 and 16 channels, respectively, followed by fully connected layers for LAI regression.

All five models used the same dataset and five predictors. The initial model comparison used one random 70:30 split, with 341 observations for training and 147 for testing. To quantify split-related uncertainty, RF and SVR were evaluated across 100 stage-stratified 70:30 splits and results are reported as mean ± standard deviation [25,26]. These resamples characterize performance within the observed data distribution rather than independent external validation because repeated locations and stages may occur in both subsets. Leave-one-plot-out validation across the 13 fixed plot groups assessed spatial/group transferability, and leave-one-growth-stage-out testing assessed temporal transferability.

For leave-one-growth-stage-out validation, observations from one growth stage were held out for testing and observations from the remaining three stages were used for training. The procedure was repeated for jointing, tasseling, silking, and grain filling with the same fixed RF and SVR settings. This group-based validation design evaluates the ability to predict a temporal group not represented in the training data [27].

RF-based feature ablation was used to compare three predictor settings: vegetation indices only, texture features only, and the selected five-feature spectral–texture combination. Each setting was evaluated with the same 100 stage-stratified splits. The paired R2 difference between the five-feature and vegetation-index-only settings was calculated across splits with a 95% confidence interval.

Model performance was assessed using the coefficient of determination (R2), root mean square error (RMSE), and relative root mean square error (RRMSE = RMSE/mean observed LAI). The single split served as the benchmark for comparison with the original manuscript. Repeated random splitting quantified internal uncertainty, leave-one-plot-out validation assessed transfer among the observed plot groups, leave-one-growth-stage-out validation assessed temporal transferability, and feature ablation assessed the relative contribution of vegetation indices and texture metrics.

## 3. Results

### 3.1. Feature Selection

Feature screening retained MCARI1_GOSAVI, GSAVI, MCARI1_MRETVI, NIR_HOM, and NIR_ENT for maize LAI estimation. The first three variables are vegetation indices and the latter two are NIR texture metrics. Together, they represent the canopy spectral response and image spatial structure. Their relationships with the measured LAI are shown in Figure 2.

The selected variables differed in both the direction and magnitude of their correlations with LAI (Figure 2). MCARI1_GOSAVI and MCARI1_MRETVI were positively correlated with LAI, whereas GSAVI and NIR_HOM were negatively correlated; NIR_ENT was positively correlated. Several predictors also shared strong intercorrelations, particularly NIR_HOM and NIR_ENT (r = −0.982). VIFs ranged from 10.51 to 31.54, indicating substantial shared information. The set should therefore be interpreted as a compact predictor combination, and individual importance rankings should not be read as independent mechanistic effects.

In the RF SHAP analysis, MCARI1_GOSAVI had the largest mean absolute contribution, followed by NIR_HOM, GSAVI, NIR_ENT, and MCARI1_MRETVI (Figure 3). Held-out permutation importance gave the same broad result for RF and SVR: the vegetation-index variables contributed more predictive information than the texture variables. Because NIR_HOM and NIR_ENT were highly correlated, their individual rankings are interpreted cautiously; together, they represent complementary canopy structure information.

The final predictor set comprised three vegetation indices and two NIR texture metrics. These five variables were carried forward for model evaluation and spatial mapping.

### 3.2. Accuracy Comparison Among Models

Table 2 summarizes the single-split accuracy of the five models using the selected spectral–texture predictors. The benchmark used 341 observations for training and 147 for testing. Results from the repeated and transferability analyses are reported separately in Table 3.

All five models reproduced the overall LAI variation in the single-split benchmark (Table 2). MLP gave the highest R^2^ (0.896) and lowest RMSE (0.240; RRMSE = 0.079), while RF was close behind (R^2^ = 0.889, RMSE = 0.249, RRMSE = 0.082). XGB and SVR produced similar R^2^ values of 0.877 and 0.875, respectively. The 1D-CNN performed less well, with R^2^ = 0.827, RMSE = 0.310, and RRMSE = 0.103. These values come from one random partition and should be interpreted as internal agreement within the current dataset, not as a definitive model ranking or evidence of external generalization.

RF was selected for mapping on the basis of more than its single-split score. It was further examined through repeated stage-stratified splitting, leave-one-plot-out testing, leave-one-growth-stage-out testing, feature ablation, and SHAP analysis. Across 100 repeated splits, RF achieved R^2^ = 0.876 ± 0.016, RMSE = 0.258 ± 0.018, and RRMSE = 0.085 ± 0.006; the corresponding SVR values were R^2^ = 0.851 ± 0.015, RMSE = 0.283 ± 0.015, and RRMSE = 0.093 ± 0.005 (Table 3). RF was retained as the reference mapping model because its uncertainty, transferability, and feature contribution were explicitly examined. The pure MLP map is included for comparison; we do not infer that RF is universally more accurate than MLP from one split.

### 3.3. Leave-One-Growth-Stage-Out Validation

Random splitting evaluates performance within the observed sample distribution, but observations from the same growth stage can appear in both the training and testing sets. This can produce optimistic estimates of temporal transferability. We therefore held out one entire growth stage at a time for testing and trained the models on the remaining three stages (Table 4).

RF performance differed substantially among the held-out stages (Table 4). Its R^2^ was −0.542 for jointing, 0.137 for tasseling, 0.441 for silking, and 0.191 for grain filling; the pooled R^2^ across all held-out predictions was 0.746. SVR was particularly unstable when jointing was held out and had a pooled R^2^ of 0.147. Thus, good random-split accuracy did not guarantee prediction of a stage absent from training, especially where canopy structure and the LAI range differed among stages.

Relative to repeated random splitting, leave-one-growth-stage-out validation showed much weaker transferability. RF was more stable than SVR, but the negative jointing-stage R^2^ shows that the present dataset does not support reliable stage-independent prediction over the full growing season. This is a limitation of the current model rather than evidence of broad temporal generalization. RF was nevertheless retained for mapping because it performed best in repeated internal analysis and it permits feature interpretation.

### 3.4. RF-Based Feature Ablation Analysis

RF-based feature ablation compared vegetation indices only, texture features only, and the selected spectral–texture combination (Table 5).

Across the 100 stage-stratified splits, RF with the three vegetation indices achieved R^2^ = 0.866 ± 0.016, RMSE = 0.268 ± 0.017, and RRMSE = 0.088 ± 0.006. The texture-only setting was much less accurate (R^2^ = 0.480 ± 0.041, RMSE = 0.529 ± 0.027, RRMSE = 0.173 ± 0.009), indicating that vegetation indices provided most of the predictive information in this dataset.

Adding NIR_HOM and NIR_ENT to the three vegetation indices gave R^2^ = 0.876 ± 0.016, RMSE = 0.258 ± 0.018, and RRMSE = 0.085 ± 0.006. The paired R^2^ gain over vegetation indices alone was 0.010, with a 95% confidence interval of 0.009–0.011 across the 100 splits. The improvement was consistent but small, so the texture metrics are interpreted as complementary predictors rather than the main source of accuracy.

### 3.5. Accuracy Analysis of Machine Learning Models

Figure 4 compares measured and predicted LAI for RF, SVR, and XGB. All three models followed the overall LAI gradient, showing that the selected spectral–texture predictors captured the main variation represented in the dataset.

RF predictions were concentrated relatively close to the 1:1 line (Figure 4a). The fitted relationship was y = 0.851x + 0.411, with R^2^ = 0.889, RMSE = 0.249, and RRMSE = 0.082. Some underestimation remained at the upper end of the LAI range.

SVR predictions were more dispersed than RF predictions (Figure 4b). The fitted relationship was y = 0.816x + 0.530, with R^2^ = 0.875, RMSE = 0.264, and RRMSE = 0.087. The pattern suggests greater underestimation at medium-to-high LAI and slight overestimation at lower LAI.

XGB followed the measured LAI gradient but showed greater scatter at the lower and upper ends of the range (Figure 4c). It achieved R^2^ = 0.877, RMSE = 0.261, and RRMSE = 0.087; the fitted relationship was y = 0.832x + 0.464, indicating modest underestimation at high LAI. XGB is therefore treated as a single-split benchmark rather than the selected mapping model. Across the available internal tests, RF had the most complete validation evidence. The stage-holdout results also show that temporal transferability remains the main limitation. The RF maps are consequently interpreted as site-specific canopy-monitoring outputs, not as treatment-effect evidence or universally applicable operational products.

### 3.6. Accuracy Analysis of Deep Learning Models

MLP and 1D-CNN were evaluated with the same single 70:30 split used for the machine learning models. Figure 5 compares their measured and predicted LAI values. MLP was close to the 1:1 line and achieved R^2^ = 0.896, RMSE = 0.240, and RRMSE = 0.079. Under the same split, 1D-CNN achieved R^2^ = 0.827, RMSE = 0.310, and RRMSE = 0.103.

The convolutional structure did not improve performance for the five structured spectral–texture predictors used here. Because the 1D-CNN was not trained on image patches or spatial neighborhoods, it is best viewed as a neural network benchmark. As with the other models, its single-split result reflects internal agreement within this dataset and does not demonstrate external generalization or universal model superiority.

### 3.7. Spatial Distribution of LAI Estimation Results

RF was used as the reference model for spatial mapping, with pure MLP retained for direct comparison (Figure 6). Both models reproduced similar broad spatial patterns across the four acquisition dates. The maps were used to describe field-scale canopy conditions; model accuracy and transferability were assessed from the point-based validation data rather than from visual map comparison.

LAI was generally lower at jointing and higher at tasseling, silking, and grain filling, consistent with canopy development over the observation period. Both models also showed localized high- and low-LAI zones within the fixed field layout.

Spatial variation occurred both among and within the fixed plot groups, and the strip-shaped field layout remained evident in the maps. Each management background, however, was represented by one plot and several plots received additional nitrogen. The observed contrasts are therefore described as canopy heterogeneity under the existing field platform, not as statistical evidence of independent tillage, straw-return, or nitrogen effects.

RF and MLP showed similar broad high- and low-LAI patterns. RF retained more local contrast, whereas MLP produced a smoother surface. This visual difference is not an independent accuracy comparison; interpretation is limited to site-specific mapping under the stated experimental constraints.

### 3.8. Frequency Distribution of LAI Across Growth Stages

Figure 7 summarizes the map-wide distributions of LAI estimated by RF and pure MLP. Both models reproduced the same seasonal progression, although MLP consistently returned a higher central LAI level. At jointing, the RF distribution had a mean of 2.702 and a standard deviation of 0.055, with the 5th–95th percentile interval spanning 2.622–2.798; its modal value was 2.700. The corresponding MLP values were 2.881, 0.051, 2.803–2.964, and 2.880, respectively. Thus, the difference in mean mapped LAI between MLP and RF was 0.179 at this early stage.

At tasseling, the RF and MLP means increased to 3.199 and 3.275, respectively, and the difference between the two models decreased to 0.076. Their modal values were 3.205 for RF and 3.270 for MLP. The central 90% of RF estimates ranged from 3.021 to 3.342, compared with 3.135–3.418 for MLP. At silking, the two distributions shifted further upward, with means of 3.581 for RF and 3.688 for MLP and modal values of 3.560 and 3.675. Grain filling retained the high-LAI pattern: the RF and MLP means were 3.622 and 3.732, and the modal values were 3.595 and 3.720, respectively.

Spatial dispersion increased as the canopy developed. For RF, the 5th–95th percentile width increased from 0.177 at jointing to 0.322 at tasseling, 0.416 at silking, and 0.549 at grain filling. The corresponding MLP widths were 0.161, 0.283, 0.429, and 0.564. The two models therefore agree on the temporal progression and the increasing spread of mapped LAI, whereas the differences in peak shape and dispersion varied with growth stage. MLP nevertheless remained centered at higher LAI throughout the observation period. These values describe the properties of the mapped surfaces; they do not constitute an additional accuracy assessment, which is based on the held-out validation data.

### 3.9. Field-Scale LAI Spatial Heterogeneity Based on RF Estimation

RF was used for the plot-level description because it served as the reference mapping model and retained the local spatial detail visible in Figure 6. The distribution curves in Figure 8 shifted rightward from jointing to silking for all 13 fixed plot groups. Figure 9 quantifies the same pattern. The mean of the 13 plot-level RF estimates was 2.708 at jointing, with a standard deviation of 0.033 among plot means and a range of 2.675–2.787. At tasseling, the average plot mean increased to 3.196 (SD = 0.030; range = 3.156–3.257).

The rise continued to silking, when the average plot mean reached 3.583 (SD = 0.031; range = 3.522–3.632). At grain filling, the average remained high at 3.639, while the range widened to 3.554–3.722 and the standard deviation among plot means increased to 0.051. Accordingly, the between-plot spread was largest at grain filling, even though the overall mean changed little after silking. Figure 8 complements these mean values by showing that the position and width of the pixel-level distributions also differed among fixed plot groups, particularly during the two late observations.

Across the 13 fixed plot groups, the average increase in mean LAI was 0.488 from jointing to tasseling and 0.387 from tasseling to silking. The subsequent mean change from silking to grain filling was only 0.057. At the individual plot level, the first two increases ranged from 0.424 to 0.516 and from 0.335 to 0.434, respectively, whereas the final-stage change ranged from −0.001 to 0.096. Thus, most of the seasonal increase occurred before or by silking, while grain filling was characterized by a high but comparatively stable mean LAI. Figure 8 and Figure 9 provide complementary descriptions of this heterogeneity: Figure 8 shows within-plot pixel distributions and Figure 9 summarizes plot-level mean values.

The standard deviations and ranges reported above describe variation among the 13 fixed plot estimates; they are not standard errors from independent treatment replicates. Each management background was represented by one fixed plot, and the groups labeled with -N also differed in nitrogen input. The observed differences are therefore presented as field-scale canopy heterogeneity and cannot be used to attribute LAI responses separately to tillage, straw return, or nitrogen addition.

## 4. Discussion

### 4.1. Contribution of Spectral–Texture Features to LAI Estimation

The final set contained three vegetation indices and two NIR texture metrics. Repeated feature ablation showed that vegetation indices supplied most of the predictive information. The texture metrics provided a small but consistent improvement in repeated random validation. Because NIR_HOM and NIR_ENT were strongly correlated and VIFs were high, their individual effects should not be interpreted independently.

NIR_HOM and NIR_ENT added information on canopy spatial structure to the spectral predictors. Previous studies likewise show that UAV-derived vegetation indices and GLCM texture metrics can be combined for LAI estimation, while texture alone is generally less effective than vegetation indices [9,10,11,12,13]. The present results follow that pattern. Given the correlation among predictors, the modest gain from the combined set is interpreted as improved prediction rather than evidence that either texture metric independently controls LAI.

### 4.2. Model Validation and Stability

The high scores from the single random split were accompanied by split-related uncertainty in the repeated analysis. Across 100 stage-stratified splits, RF achieved R^2^ = 0.876 ± 0.016 and SVR achieved R^2^ = 0.851 ± 0.015. Leave-one-plot-out validation yielded R^2^ = 0.878 ± 0.062 for RF and 0.853 ± 0.050 for SVR, suggesting that the observed feature relationships were reasonably stable among the plot groups at this site. These are internal validation results because all observations came from one field and one growing season.

Leave-one-growth-stage-out validation was the most stringent internal test. RF R^2^ ranged from −0.542 to 0.441 across the four held-out stages, with a pooled R^2^ of 0.746. SVR had a pooled R^2^ of 0.147 and performed particularly poorly when jointing was excluded from training. Models trained on mixed growth stages may therefore not transfer reliably to an unrepresented stage, even when random-split and plot-holdout results are favorable. Independent validation across years, locations, cultivars, and management conditions is needed before operational use.

### 4.3. Field-Scale LAI Spatial Heterogeneity

The RF and pure-MLP maps showed lower LAI at jointing and predominantly higher LAI classes at the following three observation dates, consistent with canopy expansion during the monitoring period. UAV imagery combined with machine learning can provide non-destructive, high-resolution information on maize canopy development and field-scale spatial variability [2,3,17,18].

Local differences in the maps may reflect management background, baseline soil conditions, water status, canopy density, and additional nitrogen application. High-resolution LAI maps can help identify low-vigor areas and provide information for future management zoning [2,3,17,18]. This work did not test variable-rate fertilization or irrigation and did not generate prescription maps. The outputs should therefore be used as canopy-monitoring information rather than as a complete precision-management decision system.

### 4.4. Uncertainties and Future Work

Several limitations should be considered. The experiment covered one site and one growing season, and the 13 management backgrounds were not independently replicated randomized treatment units. The 122 locations were revisited at four stages, so the 488 observations represent repeated temporal measurements rather than 488 independent experimental replicates. Applying the models to other years, regions, cultivars, or management conditions requires external validation. The selected predictors were also highly collinear (VIF 10.51–31.54), limiting mechanistic interpretation of individual importance rankings despite their utility for prediction. In addition, independent orthomosaic RMSE data were unavailable, and quadrat geolocation, plot boundary delineation, and exclusion of shadows and bare soil may have affected the spatial maps. Future work should combine randomized replicated field designs with independent geometric check points, larger ground samples, multi-year and multi-location observations, and external model testing before deriving management prescriptions. Evaluating treatment effects will additionally require consistent fertilization treatments, independent replication, and coordinated UAV, ground-LAI, yield, and soil water measurements.

## 5. Conclusions

1. This work developed a field-scale approach for maize LAI estimation from UAV multispectral imagery and synchronous ground measurements. It focused on a compact spectral–texture predictor set, model validation, feature ablation, and descriptive spatial patterns among fixed plots. The main findings are as follows:
2. MCARI1_GOSAVI, GSAVI, MCARI1_MRETVI, NIR_HOM, and NIR_ENT formed a compact predictor set for this dataset. Although all five variables were associated with LAI, they were also correlated. Their importance is therefore interpreted as predictive contribution rather than independent mechanism.
3. RF achieved R^2^ = 0.889, RMSE = 0.249, and RRMSE = 0.082 in the original single random split. Across 100 repeated stage-stratified splits, RF achieved R^2^ = 0.876 ± 0.016, RMSE = 0.258 ± 0.018, and RRMSE = 0.085 ± 0.006. Leave-one-plot-out validation gave R^2^ = 0.878 ± 0.062. In contrast, leave-one-growth-stage-out testing gave a pooled R^2^ of 0.746, with stage-specific values from −0.542 to 0.441. These results support internal, site-specific monitoring but not external generalization.
4. Feature ablation identified vegetation indices as the primary information source for maize LAI estimation. Adding the two NIR texture metrics increased R^2^ by 0.010 relative to vegetation indices alone across the 100 paired splits. This modest gain supports treating NIR texture as complementary canopy structure information rather than the main source of predictive accuracy.
5. RF-based maps showed lower LAI at jointing and higher LAI classes later in the season. RF and pure MLP produced similar broad spatial patterns, but the field design does not support interpreting mapped contrasts among plot groups as treatment effects. UAV multispectral imagery can provide high-resolution information for maize canopy monitoring and spatial variability assessment; management prescriptions still require independently replicated experiments and external validation.

## Figures and Tables

**Figure 1:**
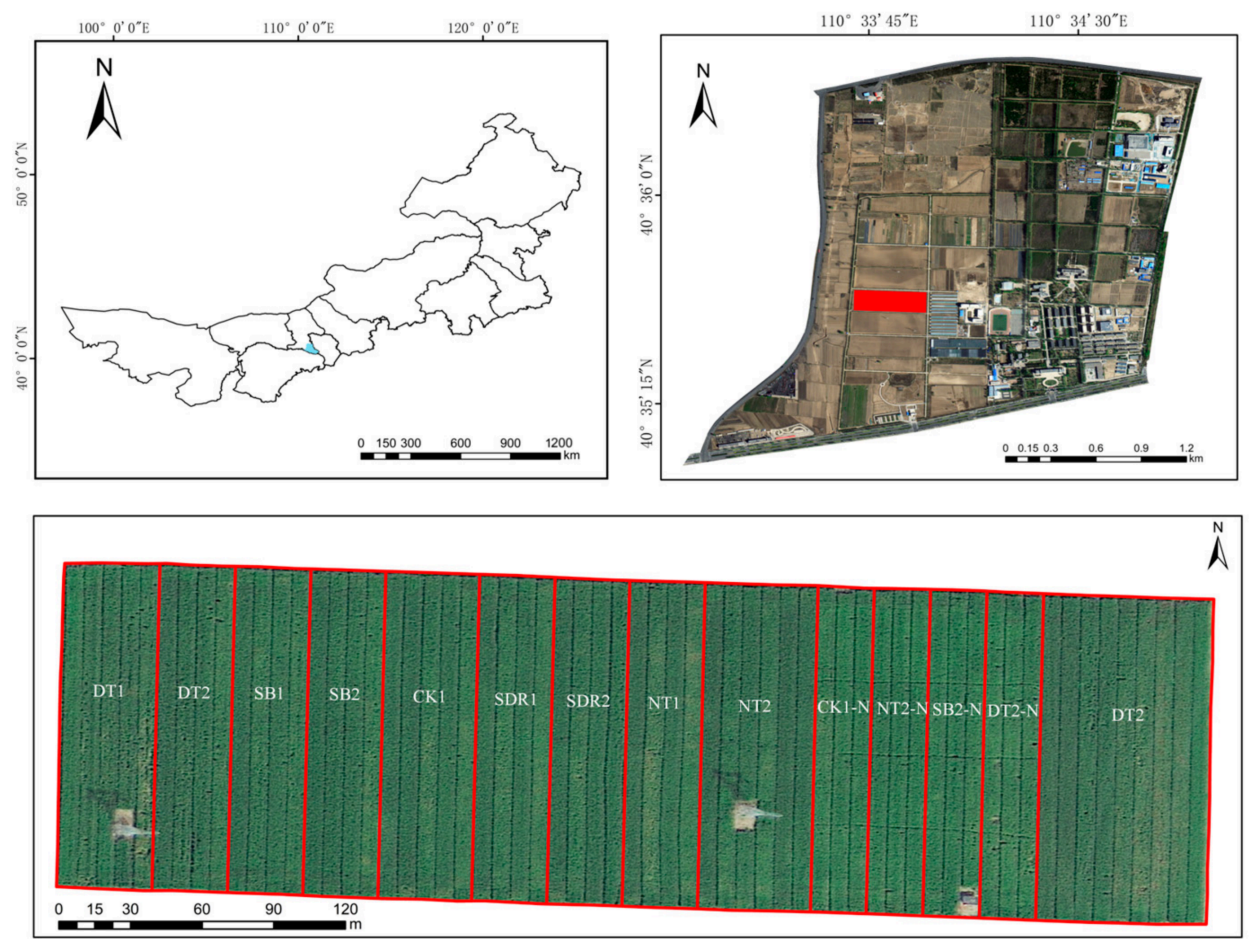
Overview of the study area and fixed experimental plots.

**Figure 2:**
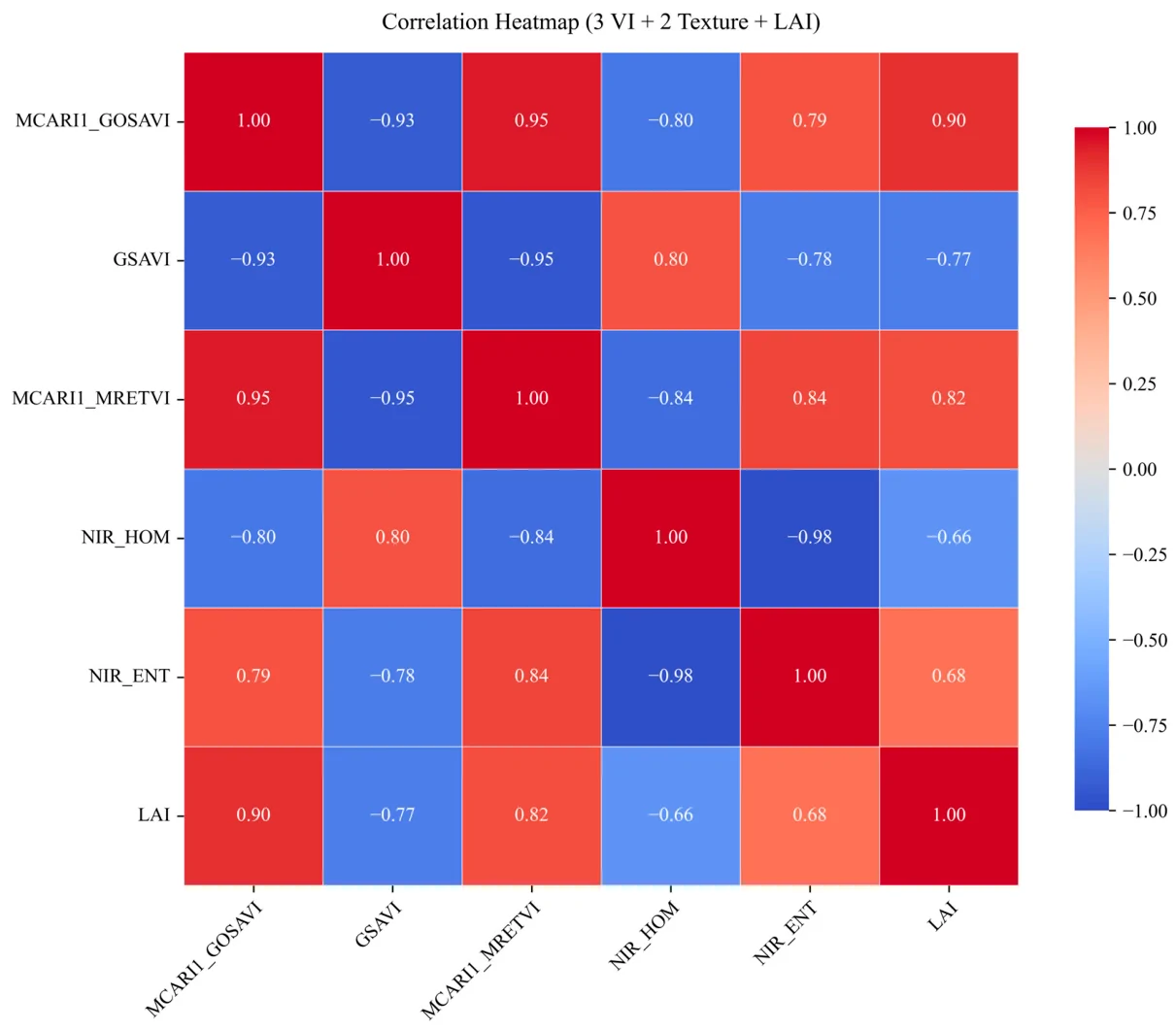
Correlation heatmap of the selected feature combination and leaf area index.

**Figure 3:**
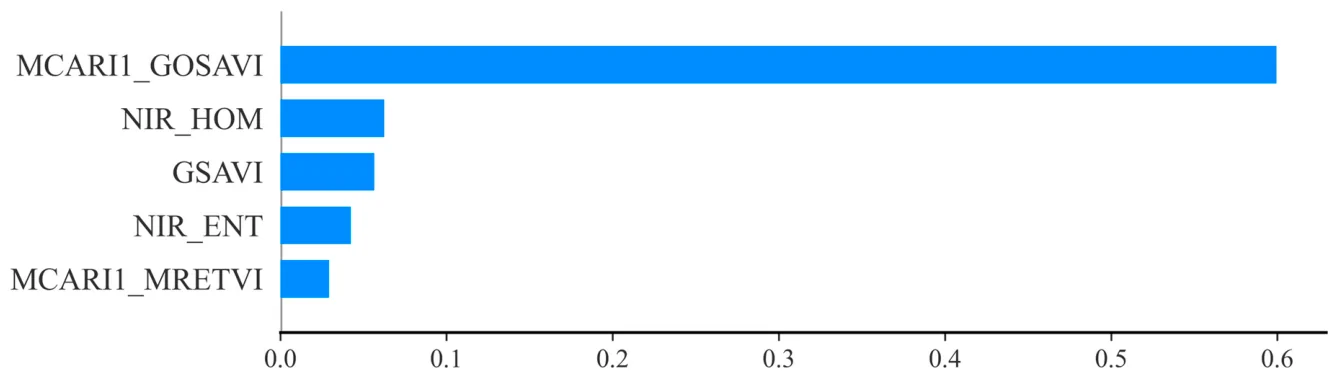
SHAP contribution analysis of the selected feature combination based on the RF model.

**Figure 4:**
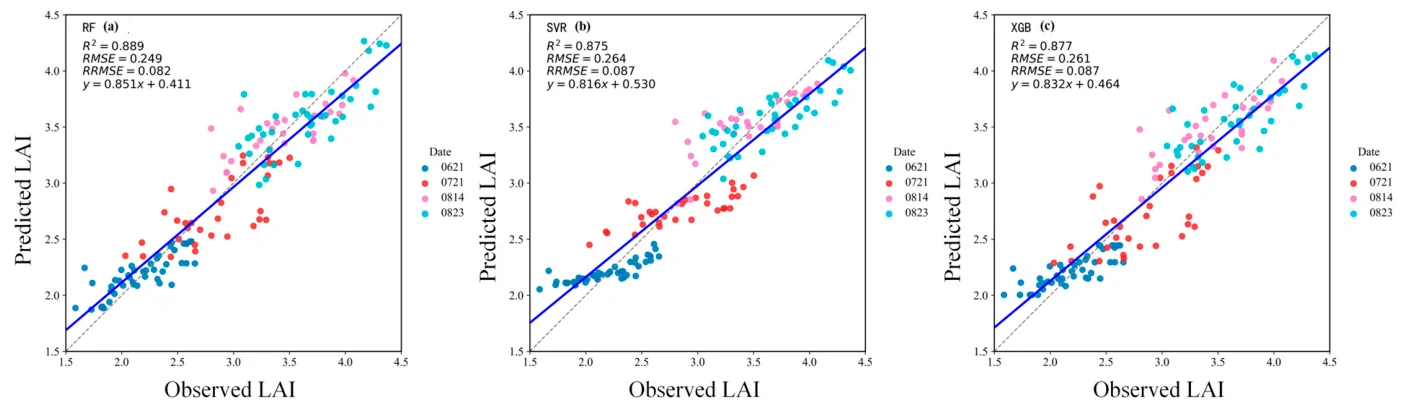
Relationship between measured and predicted LAI values based on different machine learning models: (**a**) RF; (**b**) SVR; and (**c**) XGB. The blue solid line represents the fitted linear regression line, while the gray dashed line denotes the 1:1 reference line.

**Figure 5:**
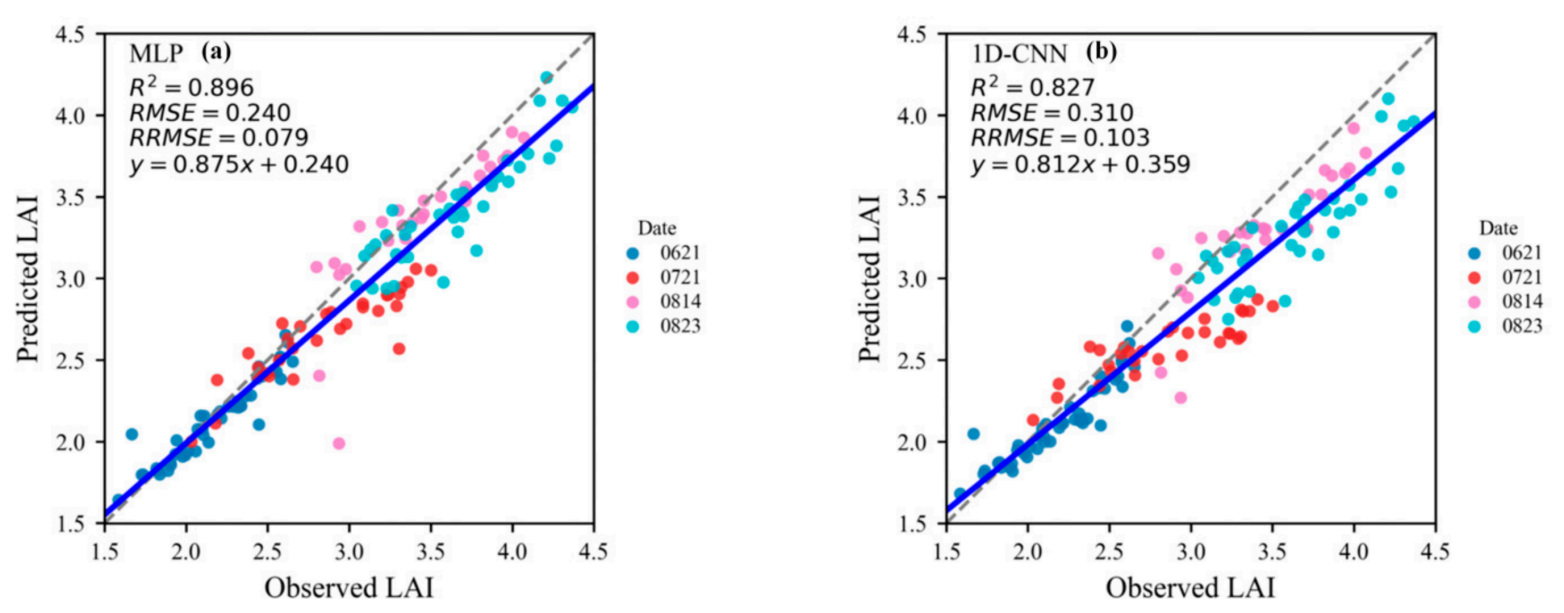
Relationships between measured and predicted LAI values for the deep learning models: (**a**) MLP; and (**b**) 1D-CNN. The blue solid line represents the fitted linear regression line, while the gray dashed line denotes the 1:1 reference line.

**Figure 6:**
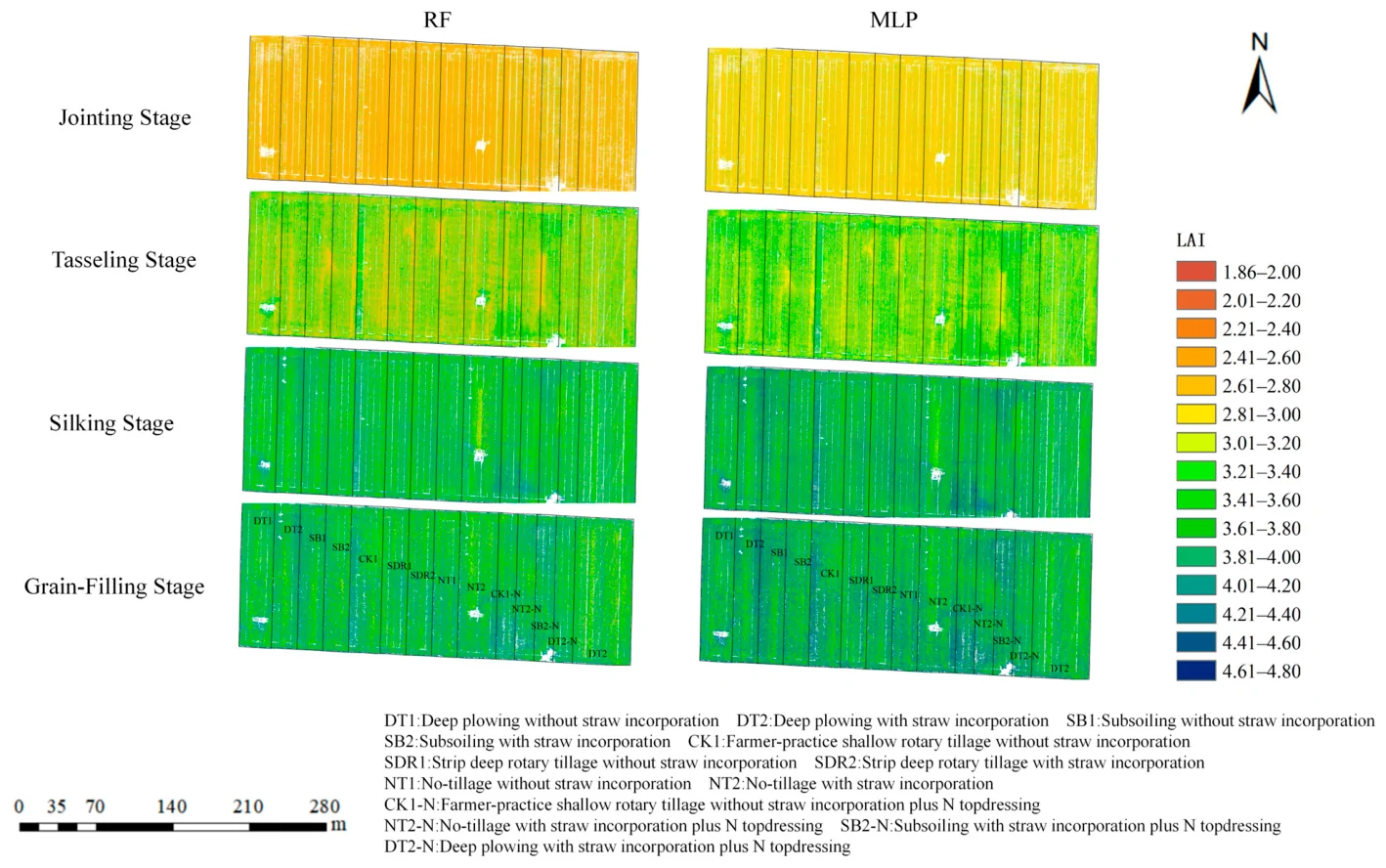
Spatial distribution of maize LAI estimated by the RF and pure MLP models at four growth stages. The maps provide a descriptive comparison of canopy conditions; differences among fixed plots do not represent independently replicated treatment effects.

**Figure 7:**
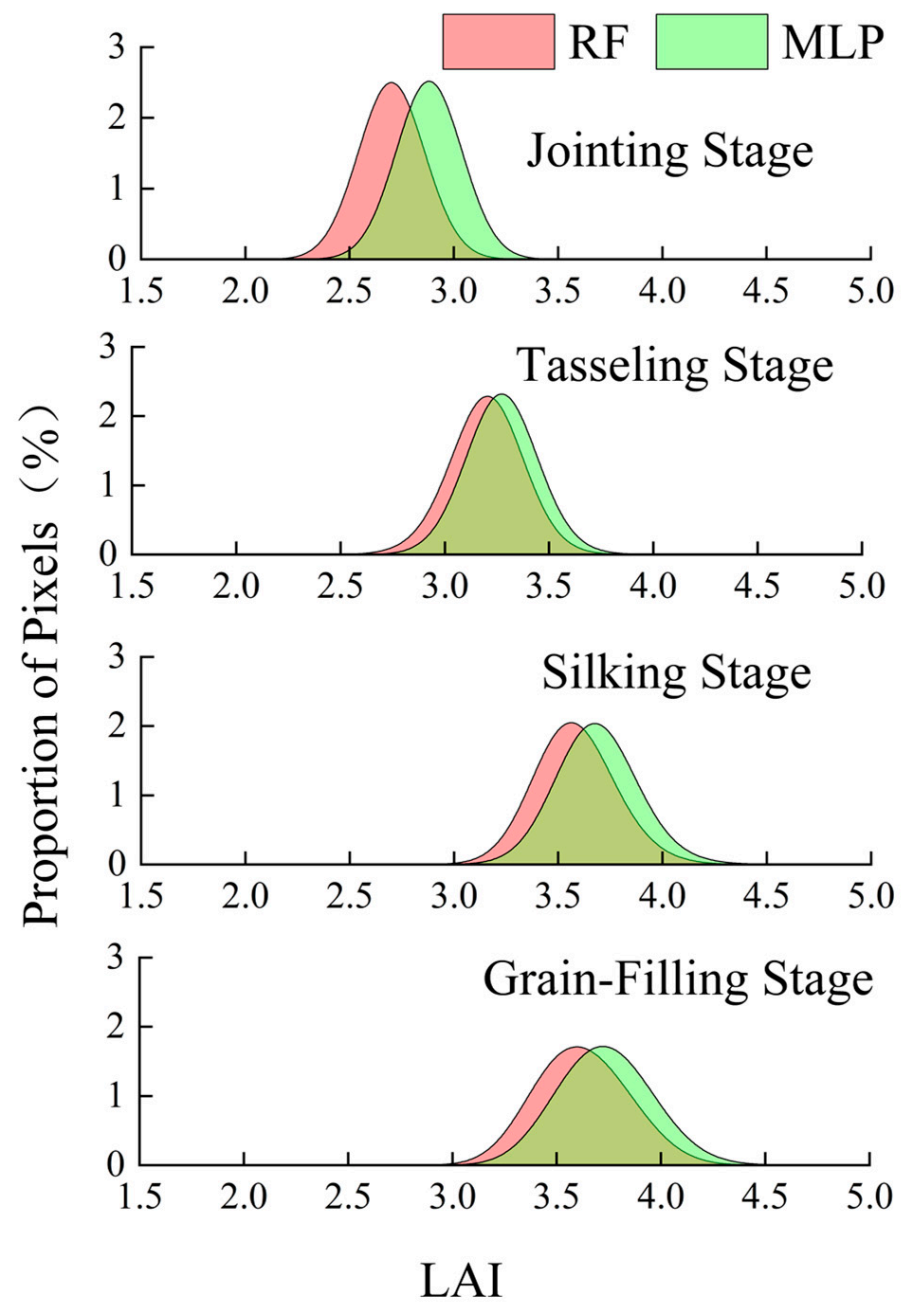
Pixel-level LAI frequency distributions estimated by the RF and pure MLP models across four growth stages.

**Figure 8:**
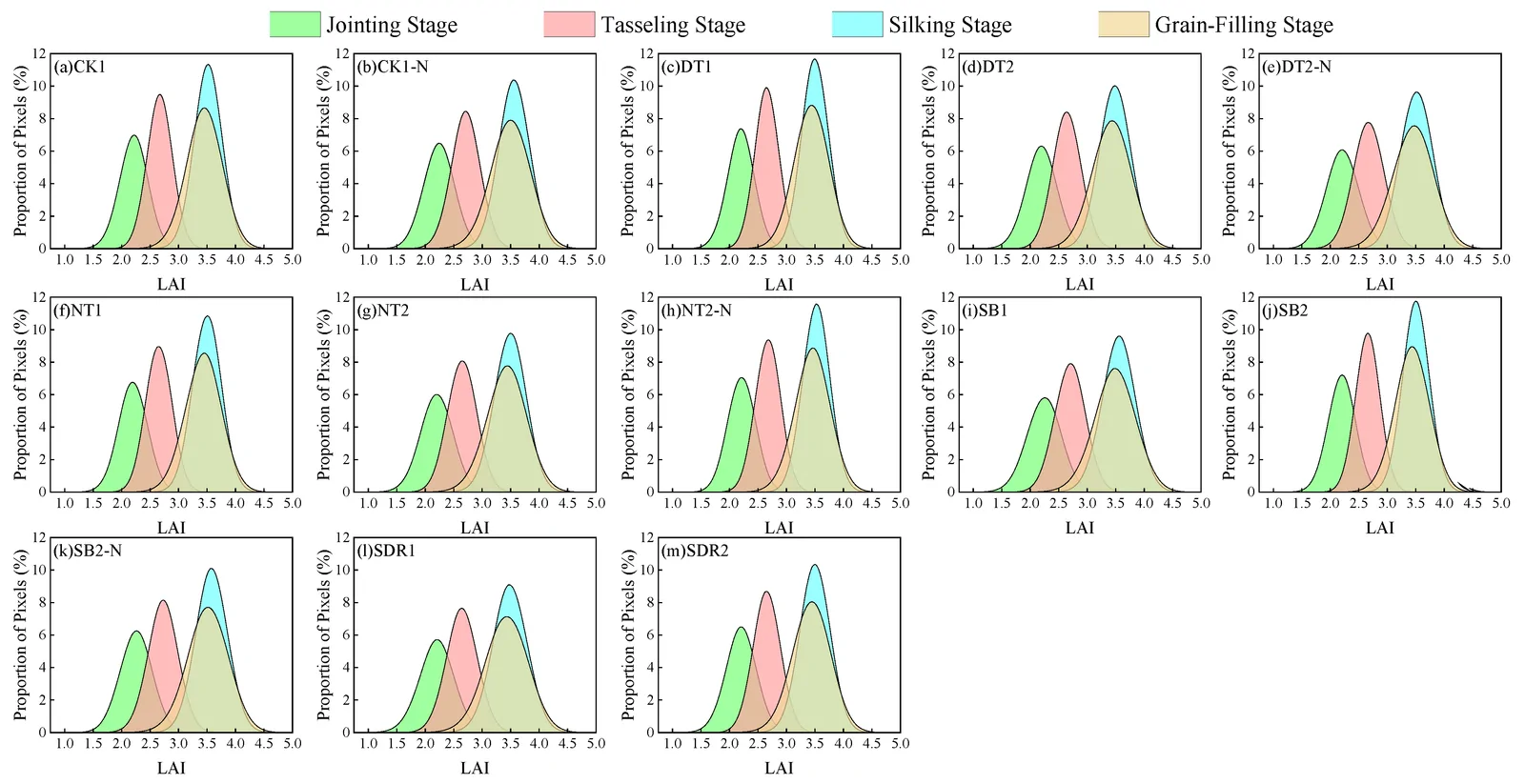
Pixel-level distributions of RF-estimated LAI within the 13 fixed plot groups across four growth stages.

**Figure 9:**
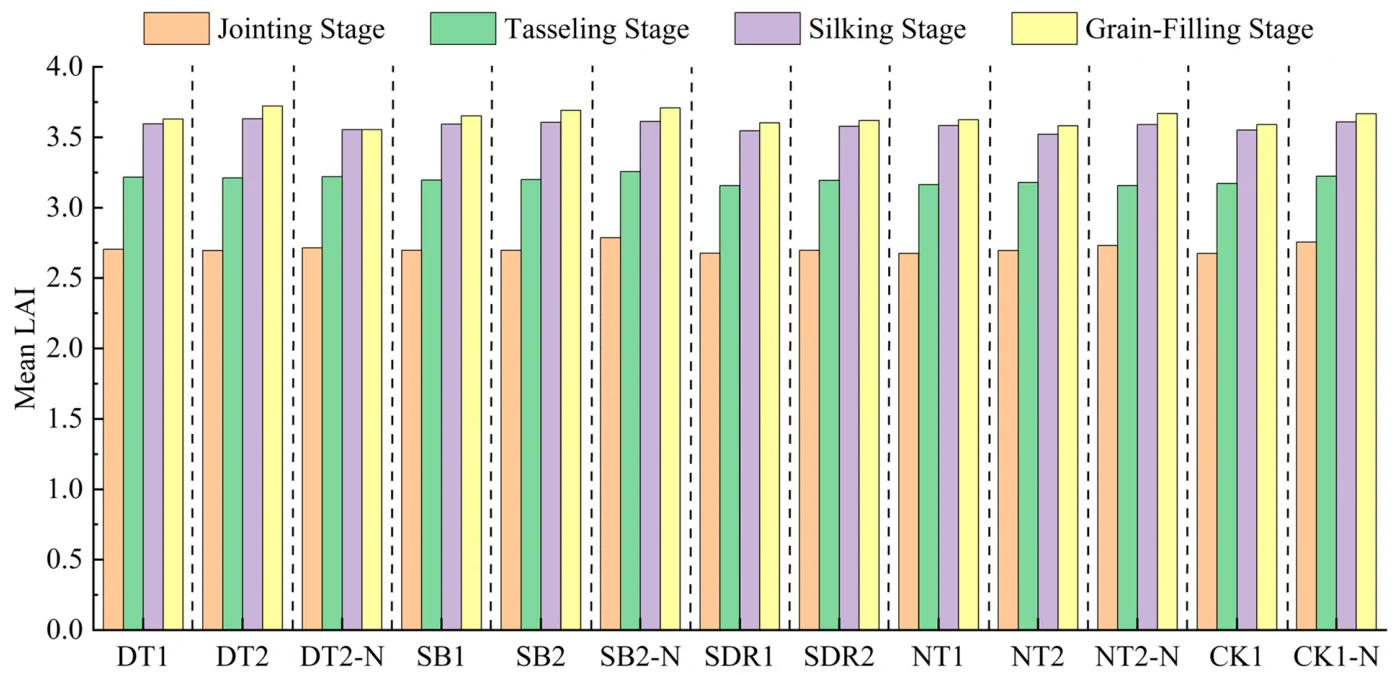
Mean maize LAI estimated by the RF model in fixed plots at different growth stages. The vertical dashed lines separate adjacent tillage-treatment plots.

**Table 1:** Categories and representative vegetation indices.

Category	Representative Indices	Primary Function
Soil-adjusted indices	GSAVI, SAVI, OSAVI, GOSAVI, RESAVI	Reduce soil background effects and enhance canopy greenness response
Red-edge-sensitive indices	RECI, MTCI, LCI, RETVI, MRETVI	Enhance responses to chlorophyll status and medium-to-high canopy cover
Combined enhancement indices	MCARI1_GOSAVI, MCARI1_MRETVI, MCARI2_GOSAVI, MCARI2_REOSAVI, MTCARI_MRETVI	Integrate absorption, red-edge, and soil background information
Normalized-difference or ratio indices	NDVI, GNDVI, NDREI, GRVI, RDVI	Describe canopy greenness and cover
Additional indices	GDVI, PVI, WDVI, EXR	Provide supplementary spectral information

**Table 2:** Comparison of maize LAI estimation accuracy among different models.

Model	R^2^	RMSE	RRMSE
RF	0.889	0.249	0.082
SVR	0.875	0.264	0.087
XGB	0.877	0.261	0.087
1D-CNN	0.827	0.310	0.103
MLP	0.896	0.240	0.079

**Table 3:** Robust internal validation of RF and SVR using the confirmed LAI-Z-SJ dataset.

Validation Strategy	RF	SVR
Single 70:30 split	R^2^ 0.889; RMSE 0.249; RRMSE 0.082	R^2^ 0.875; RMSE 0.264; RRMSE 0.087
100 repeated stage-stratified splits	R^2^ 0.876 ± 0.016; RMSE 0.258 ± 0.018; RRMSE 0.085 ± 0.006	R^2^ 0.851 ± 0.015; RMSE 0.283 ± 0.015; RRMSE 0.093 ± 0.005
Leave-one-plot-out (13 plots)	R^2^ 0.878 ± 0.062; RMSE 0.245 ± 0.044; RRMSE 0.080 ± 0.016	R^2^ 0.853 ± 0.050; RMSE 0.273 ± 0.033; RRMSE 0.089 ± 0.012

**Table 4:** Per-stage accuracy of maize LAI under leave-one-growth-stage-out validation using the fixed RF and SVR settings.

Held-Out Stage	RF R^2^	RF RMSE	RF RRMSE	SVR R^2^	SVR RMSE	SVR RRMSE
Jointing	−0.542	0.362	0.167	−15.537	1.185	0.546
Tasseling	0.137	0.425	0.152	0.270	0.391	0.140
Silking	0.441	0.261	0.076	0.346	0.282	0.082
Grain filling	0.191	0.411	0.109	0.017	0.453	0.120
Pooled held-out predictions	0.746	0.370	0.121	0.147	0.679	0.222

**Table 5:** Repeated RF ablation analysis of spectral and texture feature combinations.

Input Feature Combination	Number of Features	R^2^ (Mean ± SD)	RMSE (Mean ± SD)	RRMSE (Mean ± SD)
Vegetation indices only	3	0.866 ± 0.016	0.268 ± 0.017	0.088 ± 0.006
Texture features only	2	0.480 ± 0.041	0.529 ± 0.027	0.173 ± 0.009
Selected spectral–texture features	5	0.876 ± 0.016	0.258 ± 0.018	0.085 ± 0.006

## Data Availability

The original contributions presented in this study are included in the article.
